# The Personal Trait of Spiritual Growth Is Correlated With the White Matter Integrity of the Brain

**DOI:** 10.3389/fnhum.2022.890160

**Published:** 2022-05-12

**Authors:** Masahiro Fujino, Keita Watanabe, Yoshinori Yamakawa

**Affiliations:** ^1^Open Innovation Institute, Kyoto University, Kyoto, Japan; ^2^Institute of Innovative Research, Tokyo Institute of Technology, Tokyo, Japan; ^3^Academic and Industrial Innovation, Kobe University, Hyogo, Japan; ^4^ImPACT Program of Council for Science, Technology, and Innovation (Cabinet Office, Government of Japan), Tokyo, Japan; ^5^Brain Impact General Incorporated Association, Kyoto, Japan

**Keywords:** brain healthcare quotient, diffusion tensor imaging, fractional anisotropy, white matter integrity, voxel-based morphometry, gray matter volume, spiritual growth, personal trait

## Abstract

Determining the relationship between the entire brain structure and individual differences is important in extending healthy life expectancy, which can be affected by brain atrophy. The entire brain structure has been gradually known to be correlated not only with age but also with individual differences, such as quality of life, general intelligence, and lifestyle. However, little attention has been paid to the relationship between the entire brain structure and personal traits. We herein focused on one personal trait, namely spiritual growth, and examined its relationship with the entire brain structure using two neuroimaging-derived measures, namely the gray matter Brain Healthcare Quotient (GM-BHQ), a measure of GM volume, and the fractional anisotropy Brain Healthcare Quotient (FA-BHQ), a measure of white matter (WM) integrity, in 229 healthy participants (53 female, 176 male). The results indicated no significant relationship between the GM-BHQ and spiritual growth, but there was a significant positive correlation between the FA-BHQ and spiritual growth after controlling for age, sex, and body mass index (BMI) with partial correlation analysis. Furthermore, multiple regression analysis revealed a significant positive correlation between the FA-BHQ and spiritual growth after controlling for physical characteristics, such as age, sex, and BMI, as well as other variables related to lifestyle that were collected using the Health-Promoting Lifestyle Profile. These results support the idea that there is a relationship between the entire WM brain structure and spiritual growth. Further studies are required to clarify the causal relationship between the entire WM brain structure and spiritual growth with some interventions to improve spiritual growth. Such studies will help extend healthy life expectancy from a new perspective of personal trait.

## Introduction

Life expectancy is increasing continuously and substantially; hence, extending healthy life expectancy is very important. One of the greatest causes of the gap between life expectancy and healthy life expectancy is brain atrophy (Kramer et al., 2007; Granholm et al., 2008; Henneman et al., 2009; Ahdidan et al., 2011). It has been gradually known that the entire brain structure is correlated not only with age but also with individual differences, such as quality of life (QOL), general intelligence, and lifestyle factors, such as physical activity, smoking, vegetable intake, fatigue, and stress (Nemoto et al., 2017; Kim et al., 2018; Kokubun et al., 2018; Cox et al., 2019; Kokubun and Yamakawa, 2019; Ourry et al., 2021). However, little attention has been paid to the relationship between the entire brain structure and personal traits that can advance our knowledge of brain atrophy and aid in its prevention.

This study focused on spiritual growth, a subscale of the Health-Promoting Lifestyle Profile II (HPLP II) (Walker et al., 1987; Walker and Hill-Polerecky, 1996), as a personal trait. Walker and Hill-Polerecky (1996) suggested that spiritual growth focuses on the development of inner resources and is achieved through “transcending,” “connecting,” and “developing.” Transcending enables us to have new options for becoming something more by going beyond who we are and helps us develop inner peace. Connecting is the feeling of harmony, wholeness, and connection with the universe. Developing involves maximizing human potential for wellness by searching for meaning, finding a sense of purpose, and working toward one’s goals in life (Walker and Hill-Polerecky, 1996). Previous studies have shown that spiritual growth is positively correlated with age and QOL and negatively correlated with depression, anxiety, and hostility (Frame et al., 2005; Chang et al., 2010; Tyszka and Farber, 2010; Rakhshani et al., 2014). Importantly, other studies have indicated that some interventions, such as online self-esteem intervention and relaxation response practice, improve spiritual growth (Chang et al., 2010; Robinson-Whelen et al., 2020); that is, spiritual growth is a modifiable personal trait. Furthermore, there were no significant cultural differences between American and Japanese cohorts (Kemppainen et al., 2011). Thus, we can conclude that spiritual growth is related to long-term inner personal growth until the end of life and with the quality of the whole life, and there may be a relationship between the entire brain structure and spiritual growth.

We undertook a cross-sectional study, focusing on the Brain Healthcare Quotient (BHQ), an index for brain healthcare, to investigate the relationship between spiritual growth and the entire brain structure. BHQ includes two subordinate indices: the gray matter (GM)-BHQ, based on the volume of the GM of the whole brain, as assessed by voxel-based morphometry, and the fractional anisotropy (FA)-BHQ, based on the FA value of the white matter (WM) integrity of the whole brain, as assessed by diffusion tensor imaging (DTI) (Nemoto et al., 2017). The GM-BHQ is inversely correlated with age and negative wellbeing, such as obesity, fatigue, and stress (Nemoto et al., 2017; Kokubun et al., 2018) and is positively correlated with positive traits, such as curiosity, grit, and self-efficacy (Kokubun et al., 2020b). The FA-BHQ is inversely correlated with age and is positively correlated with a sense of life improvement that is assessed by two preliminary questions (“How is your life now compared to this time last year?” and “How do you think your life will be in the future?”) (Nemoto et al., 2017). As these whole-brain indices are correlated with age, wellbeing, and some personal traits, they can be useful indices to investigate the relationship between the entire brain structure and spiritual growth.

The aim of this study was to elucidate the relationship of spiritual growth with the GM-BHQ and FA-BHQ. We hypothesized that the two BHQ measures have a positive correlation with spiritual growth. In addition, we explored the relationship between the two measures and spiritual growth after controlling for variables related to lifestyle, as previous studies have also indicated some correlations between the entire brain structure and lifestyle.

## Materials and Methods

### Participants

We recruited 229 healthy participants (53 females, 176 males) aged 22–63 years (mean age = 42.4 years, *SD* = 10.9 years) in Tokyo, Japan. At the time of recruitment, potential participants with a history of neurological, psychiatric, or medical conditions were excluded. This study was approved by the ethics committees of the Tokyo Institute of Technology (approval number: 2019066) and was conducted in accordance with the guidelines and regulations of the institute. All participants provided written informed consent before participation.

### Measures

#### Body Mass Index

The body mass index (BMI) is a statistical measure of the weight of a person scaled according to height and is calculated as weight (kg)/height (m)^2^. Previous studies indicated that BMI was inversely correlated with the GM-BHQ (Nemoto et al., 2017; Kokubun et al., 2020a).

#### Spiritual Growth

We used the spiritual growth subscale of the HPLP II scale to measure spiritual growth. The HPLP II has 52 items and six subscales: spiritual growth (HPLP_SG), health responsibility (HPLP_HR), physical activity (HPLP_PA), nutrition (HPLP_N), interpersonal relations (HPLP_IR), and stress management (HPLP_SM). This scale measures health-promoting lifestyles that maintain or enhance the level of wellness, self-actualization, and fulfillment of an individual. Participants were asked to rate their agreement to items on a 4-point Likert scale. In our study, a Japanese version of the HPLP II (Wei et al., 2000) was used. The nine-item spiritual growth subscale is listed in Appendix.

### Magnetic Resonance Imaging Data Acquisition

Magnetic resonance imaging (MRI) data were collected using a 3T scanner (MAGNETOM Prisma; Siemens, Germany) with a 32-channel head array coil located at the Tokyo Institute of Technology. High-resolution structural images were acquired using a three-dimensional (3D) T1-weighted magnetization-prepared rapid-acquisition gradient echo (MP-RAGE) pulse sequence (repetition time (TR) = 1,900 ms, echo time (TE) = 2.52 ms, inversion time, 900 ms, flip angle = 9°, matrix size = 256 × 256, field of view (FOV) = 256 mm, slice thickness = 1 mm). DTI were acquired using spin-echo echo-planar imaging (SE-EPI) with generalized autocalibrating partially parallel acquisitions (GRAPPA) (TR = 14,100 ms, TE = 81 ms, flip angle = 90°, matrix size = 114 × 114, FOV = 224 mm, slice thickness = 2 mm). A baseline image (*b* = 0 s/mm^2^) and 30 different diffusion orientations were acquired with a *b* value of 1,000 s/mm^2^.

### Magnetic Resonance Imaging Data Analysis

T1-weighted images were preprocessed and analyzed using Statistical Parametric Mapping 12 (SPM12; Wellcome Trust Centre for Neuroimaging, London, United Kingdom) using MATLAB R2015b (Mathworks Inc., Sherborn, MA, United States). In the preprocessing steps, segmentation, bias correction, and spatial normalization were incorporated into a single generative model (Ashburner and Friston, 2005). First, each image was segmented into GM, WM, and cerebrospinal fluid (CSF) images using SPM12 prior probability templates. To aid segmentation by correcting for scanner-induced smooth intensity differences that varied in space, the intensity non-uniformity bias correction was applied. Second, the segmented GM images were spatially normalized using the diffeomorphic anatomical registration through exponentiated lie algebra (DARTEL) algorithm (Ashburner, 2007). A modulation step was incorporated during the normalization step to reflect the regional volume and preserve the total GM volume from before the warp. Third, all segmented and normalized images were smoothed with an 8-mm full width at a half-maximum (FWHM) Gaussian kernel. Additionally, to control for differences in the whole-brain volume across participants, proportional GM images were generated by dividing these segmented, normalized, and smoothed GM images by intracranial volume (ICV). The ICV was calculated by summing the global volumes of GM, WM, and CSF, each of which was estimated as the total number of voxels multiplied by the voxel size for each subject.

Next, the GM-BHQ for each participant was calculated using the proportion of GM. The concept of GM-BHQ calculation is similar to the intelligence quotient. Each mean value was defined as GM-BHQ 100, and each SD was defined as 15 GM-BHQ points in a sample. By this definition, 68% of the population is between GM-BHQ 85 and GM-BHQ 115, and 95% of the population is between GM-BHQ 70 and GM-BHQ 130. In this research, we used the database of Nemoto et al. (2017) as a sample to calculate the GM-BHQ. In the database, data from 144 healthy participants (64 females, 80 males; mean age = 48.4 years, *SD* = 8.1 years) were acquired using a 3T scanner (MAGNETOM Verio or MAGNETOM Prisma, Siemens, Germany) with a 32-channel head array coil at RIKEN, Kyoto University, and the University of Tokyo with the same parameters as those used by Nemoto et al. (2017). First, individual GM quotient images were calculated using the following formula: 100 + 15 × (individual proportional GM - mean)/*SD*. Second, regional GM quotients were extracted using an automated anatomical labeling (AAL) atlas (Tzourio-Mazoyer et al., 2002). Finally, the GM-BHQ was obtained by averaging across regional GM quotients.

DTI data were preprocessed and analyzed using the FMRIB Software Library (FSL) 5.0.9 (Jenkinson et al., 2012). First, motion and eddy current distortion was corrected based on each *b0* image using eddy_correct. Second, FA maps were created using dtifit. Third, FA maps were normalized to the standard Montreal Neurological Institute (MNI) space using FMRIB’s Linear Image Registration Tool (FLIRT) and FMRIB’s Non-linear Image Registration Tool (FNIRT). The resulting FA maps were smoothed using an 8-mm FWHM Gaussian kernel. Next, the FA-BHQ for each participant was calculated with normalized and smoothed FA images in the same manner as the GM-BHQ calculation, using the database of Nemoto et al. (2017) as a sample. First, individual FA quotient images were calculated using the following formula: 100 + 15 × (individual FA - mean)/*SD*. Second, regional FA quotients were extracted using Johns Hopkins University (JHU) ICBM-DTI-81 WM atlas tract labels (Mori et al., 2008) by registering the JHU FA image to MNI. Finally, the FA-BHQ was obtained by averaging across 81 regional FA quotients.

### Statistical Analysis

First, we calculated the Pearson correlation coefficients among the GM-BHQ, FA-BHQ, age, BMI, HPLP_SG, HPLP_HR, HPLP_PA, HPLP_N, HPLP_IR, and HPLP_SM, after checking the following criteria: (1) The mean is approximately equal to the median. (2) The SD is relatively small. (3) The skewness is nearly zero. (4) Kurtosis is nearly zero. (5) Each data of the QQ plot is almost on a straight line. We also calculated the Spearman correlation coefficients of sex with other variables. Second, we examined the Pearson partial correlation coefficients among the GM-BHQ, FA-BHQ, HPLP_SG, HPLP_HR, HPLP_PA, HPLP_N, HPLP_IR, and HPLP_SM, after controlling for sex, age, and BMI. Third, we performed multiple regression analysis to investigate whether there was a relationship between the entire brain structure and spiritual growth by controlling for age, sex, BMI, and variables related to lifestyle. In this analysis, we used mean-centered variables to avoid multicollinearity issues. Sex, age, and BMI were included as control variables; the dependent variables included the GM-BHQ and FA-BHQ, and the independent variables included the HPLP_SG, HPLP_HR, HPLP_PA, HPLP_N, HPLP_IR, and HPLP_SM. Furthermore, multicollinearity was checked using the variation inflation factor (VIF). The significance level was set at 0.05. All statistical analyses were performed using SPSS 28.0 (IBM Corp., Armonk, NY, United States).

## Results

**FIGURE 1:**
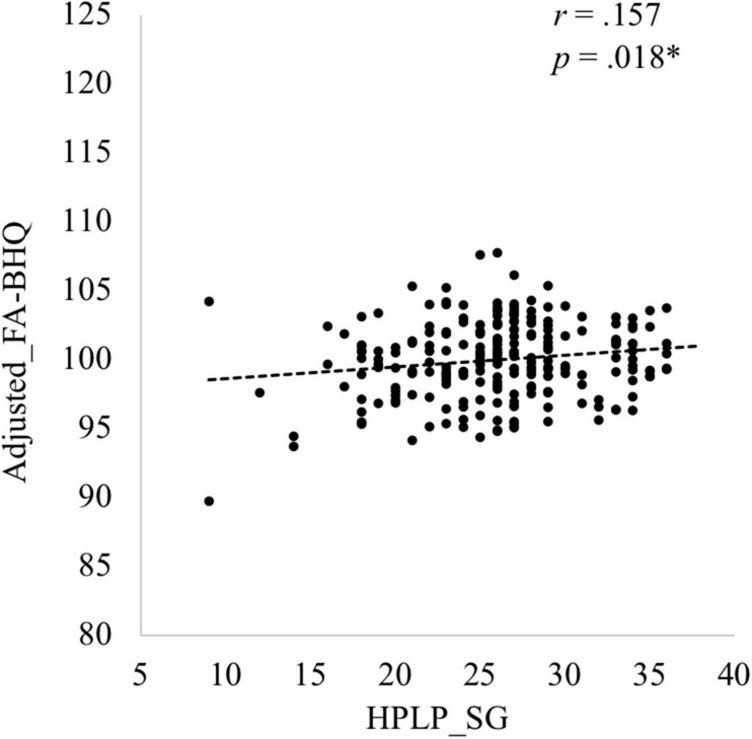
Scatter plot of the adjusted Fractional Anisotropy Brain Healthcare Quotient (FA-BHQ), adjusted for age, sex, and Body Mass Index based on the general linear model, and Health-Promoting Lifestyle Profile (HPLP) Spiritual Growth (SG).

Descriptive data of each variable are shown in Table 1. The six components of the HPLP (HPLP_SG, HPLP_HR, HPLP_PA, HPLP_N, HPLP_IR, and HPLP_SM) demonstrated moderately good internal consistency (Cronbach’s alpha = 0.88, 0.79, 0.85, 0.66, 0.79, and 0.69, respectively). The correlation coefficients among the GM-BHQ, FA-BHQ, sex, age, BMI, HPLP_SG, HPLP_HR, HPLP_PA, HPLP_N, HPLP_IR, and HPLP_SM are shown in Table 2. There is a significant correlation between the GM-BHQ and the FA-BHQ (*r* = 0.392, *p* < 0.001). Furthermore, the GM-BHQ significantly correlated with age (*r* = −0.668, *p* < 0.001), sex (*r* = 0.341, *p* < 0.001), BMI (*r* = −0.290, *p* < 0.001), HPLP_HR (*r* = −0.146, *p* = 0.027), and HPLP_N (*r* = −0.138, *p* = 0.036), and the FA-BHQ significantly correlated with age (*r* = −0.374, *p* < 0.001). The partial correlation coefficients among the GM-BHQ, FA-BHQ, HPLP_SG, HPLP_HR, HPLP_PA, HPLP_N, HPLP_IR, and HPLP_SM, after controlling for sex, age, and BMI, are shown in Table 3. There is a significant partial correlation between the GM-BHQ and the FA-BHQ (*r* = 0.191, *p* = 0.004). Furthermore, there is a significant partial correlation between the FA-BHQ and the HPLP_SG (*r* = 0.158, *p* = 0.017). The scatter plot of the adjusted FA-BHQ, which was adjusted for age, sex, and BMI based on the general linear model, and the HPLP_SG is shown in Figure 1. The result of the multiple regression analyses of the six subscales on the GM-BHQ is shown in Table 4, and that of the six subscales on the FA-BHQ is shown in Table 5. In both analyses, as all variables (age, sex, BMI, HPLP_SG, HPLP_HR, HPLP_PA, HPLP_N, HPLP_IR, and HPLP_SM) reported VIF values below 2 (VIF = 1.155, 1.258, 1.213, 1.785, 1.733, 1.593, 1.540, 1.909, and 1.590, respectively), our results did not suffer from multicollinearity. As a result, for the GM-BHQ, there is a significant coefficient of determination (*R*^2^ = 0.562, *p* < 0.001). Furthermore, there are also significant standardized partial regression coefficients of age, sex, and BMI (age: β = −0.425, *p* < 0.001; sex: β = 4.798, *p* < 0.001; BMI: β = −0.278, *p* = 0.013). Our result indicates no relationship between the GM-BHQ and the HPLP_SG. For the FA-BHQ, there is a significant coefficient of determination (*R*^2^ = 0.184, *p* < 0.001). Furthermore, there are significant standardized partial regression coefficients of age and the HPLP_SG (age: β = −0.106, *p* < 0.001; HPLP_SG: β = 0.120, *p* = 0.013). Our result indicates a relationship between the FA-BHQ and the HPLP_SG after controlling for age, sex, BMI, the HPLP_HR, HPLP_PA, HPLP_N, HPLP_IR, and HPLP_SM.

**TABLE 1:** Descriptive data.

Variable	*M*	*SD*	Range
GM-BHQ	101.75	7.48	81.22–123.62
FA-BHQ	100.71	3.03	89.67–108.73
Age	42.40	10.95	22–63
BMI	22.84	3.31	15.06–38.72
HPLP_SG	26.09	5.17	9–36
HPLP_HR	20.96	4.77	9–32
HPLP_PA	17.60	5.90	8–30
HPLP_N	23.01	4.08	13–32
HPLP_IR	26.39	4.30	14–36
HPLP_SM	22.47	3.90	12–32

*GM-BHQ, gray matter Brain Healthcare Quotient; FA-BHQ, fractional anisotropy Brain Healthcare Quotient; BMI, body mass index; HPLP, health-promoting lifestyle profile; SG, spiritual growth; HR, health responsibility; PA, physical activity; N, nutrition; IR, interpersonal relations; SM, stress management.*

**TABLE 2:** Correlation matrix.

	1	2	3	4	5	6	7	8	9	10	11
1. GM-BHQ	−	0.392***	−0.668***	0.341***	−0.29***	–0.106	−0.146*	−0.126†	−0.138*	–0.025	0.009
2. FA-BHQ		−	−0.374***	0.103	–0.097	0.117†	–0.068	0.037	–0.058	0.012	0.092
3. Age			−	–0.069	0.114†	0.066	0.231***	0.144*	0.270***	–0.024	0.024
4. Sex^a,b^				−	−0.382***	–0.033	0.169*	–0.028	0.183**	0.107	0.108
5. BMI					−	0.101	0.018	–0.040	–0.086	0.146*	0.053
6. HPLP_SG						−	0.282***	0.326***	0.233***	0.605***	0.476***
7. HPLP_HR							−	0.447***	0.518***	0.397***	0.285***
8. HPLP_PA								−	0.392***	0.269***	0.458***
9. HPLP_N									−	0.199**	0.238***
10. HPLP_IR										−	0.449***
11. HPLP_SM											−

*^a^Sex was coded as male: 1 and female: 2.*

*^b^Spearman correlation coefficients of sex with other variables were calculated.*

n = 229; ^†^p < 0.1, *p < 0.05, **p < 0.01, ***p < 0.001.

*GM-BHQ, gray matter Brain Healthcare Quotient; FA-BHQ, fractional anisotropy Brain Healthcare Quotient; BMI, body mass index; HPLP, health-promoting lifestyle profile; SG, spiritual growth; HR, health responsibility; PA, physical activity; N, nutrition; IR, interpersonal relations; SM, stress management.*

**TABLE 3:** Partial correlation matrix controlling for sex*^a^*, age, and BMI.

	1	2	3	4	5	6	7	8
1. GM-BHQ	−	0.191**	–0.077	–0.064	–0.053	–0.043	–0.081	0.002
2. FA-BHQ		−	0.158*	0.010	0.098	0.032	0.002	0.107
3. HPLP_SG			−	0.276***	0.329***	0.236***	0.607***	0.473***
4. HPLP_HR				−	0.443***	0.470***	0.400***	0.269***
5. HPLP_PA					−	0.379***	0.297***	0.472***
6. HPLP_N						−	0.211**	0.229***
7. HPLP_IR							−	0.435***
8. HPLP_SM								−

*^a^Sex was coded as male: 1 and female: 2.*

n = 229; *p < 0.05, **p < 0.01, ***p < 0.001.

*BMI, body mass index; GM-BHQ, gray matter Brain Healthcare Quotient; FA-BHQ, fractional anisotropy Brain Healthcare Quotient; HPLP, health-promoting lifestyle profile; SG, spiritual growth; HR, health responsibility; PA, physical activity; N, nutrition; IR, interpersonal relations; SM, stress management.*

**TABLE 4:** Multiple regression analysis of the HPLP factors on GM-BHQ.

	GM-BHQ	
	β	*p*-value
**Control variables**		
Age	–0.425	< 0.001***
Sex*^a^*	4.798	< 0.001***
BMI	–0.278	0.013*
**Main variables**		
HPLP_SG	–0.058	0.506
HPLP_HR	–0.027	0.768
HPLP_PA	–0.036	0.616
HPLP_N	–0.011	0.912
HPLP_IR	–0.069	0.525
HPLP_SM	0.109	0.317
*R* ^2^	0.562	< 0.001***
Adjusted *R*^2^	0.544	< 0.001***

^a^Sex was coded as male: 1 and female: 2. n = 229; *p < 0.05, ***p < 0.001. GM-BHQ, gray matter Brain Healthcare Quotient; BMI, body mass index; HPLP, health-promoting lifestyle profile; SG, spiritual growth; HR, health responsibility; PA, physical activity; N, nutrition; IR, interpersonal relations; SM, stress management.

**TABLE 5:** Multiple regression analysis of HPLP factors on FA-BHQ.

	FA-BHQ	
	β	*p*-value
**Control variables**		
Age	–0.106	< 0.001***
Sex^a^	0.543	0.270
BMI	–0.024	0.691
**Main variables**		
HPLP_SG	0.120	0.013*
HPLP_HR	–0.014	0.782
HPLP_PA	0.031	0.439
HPLP_N	–0.008	0.881
HPLP_IR	–0.108	0.072†
HPLP_SM	0.037	0.532
*R* ^2^	0.184	< 0.001***
Adjusted *R*^2^	0.151	< 0.001***

^a^Sex was coded as male: 1 and female: 2. n = 229; ^†^p < 0.1, *p < 0.05, ***p < 0.001. FA-BHQ, fractional anisotropy Brain Healthcare Quotient; BMI, body mass index; HPLP, health-promoting lifestyle profile; SG, spiritual growth; HR, health responsibility; PA, physical activity; N, nutrition; IR, interpersonal relations; SM, stress management.

## Discussion

The aim of this study was to elucidate the relationship between the entire brain structure and spiritual growth, the personal trait related to developing human potential for wellness by searching for meaning, finding a sense of purpose, and working toward goals in life (Walker and Hill-Polerecky, 1996). To achieve this aim, we used the GM-BHQ, which measures the volume of GM in the brain, and the FA-BHQ, which measures WM integrity of the brain, as the indices of the entire brain structure. We found that there was no significant relationship between the GM-BHQ and spiritual growth, but there was a significant positive correlation between the FA-BHQ and spiritual growth after controlling for variables related to physical characteristics, such as age, sex, and BMI as well as other variables related to lifestyle such as health responsibility, physical activity, nutrition, interpersonal relations, and stress management. Among the factors of the HPLP used to measure the health-promoting lifestyles that maintain or enhance the level of wellness, self-actualization, and fulfillment of the individual, spiritual growth seems to be the most related to the development of inner resources. In a previous study, the FA-BHQ was found to be correlated with a sense of life improvement as assessed using the two preliminary questions mentioned above (Nemoto et al., 2017). Our study confirmed this relationship between the FA-BHQ and a sense of life improvement with a reliable and valid questionnaire.

The FA-BHQ was calculated from the value of the WM integrity of the entire brain. The WM integrity supports the transmission efficiency of the network between brain regions (Johansen-Berg, 2010). It is considered that optimizing the speed or synchronicity of impulse transmission is important in learning and developing skills, as any complex task for achieving a goal requires the transmission of information through a series of distant cortical regions with distinct task-relevant functions (Fields, 2008; Zatorre et al., 2012). Additionally, previous studies suggested that several types of learning and training alter the WM integrity of the corresponding brain regions (Bengtsson et al., 2005; Scholz et al., 2009; Tang et al., 2010; Engvig et al., 2012; Taubert et al., 2012; Zatorre et al., 2012; Prosperini et al., 2014). Considering that spiritual growth is related to the development of inner resources, which requires several experiences, learnings, and trainings, our finding supports the idea that there is a relationship between the entire WM brain structure and spiritual growth.

In our study, we could only show that there was a correlation between the WM integrity of the entire brain and spiritual growth at one specific point in time, as we used a cross-sectional design. Considering that previous studies indicated that some interventions could improve spiritual growth (Chang et al., 2010; Robinson-Whelen et al., 2020), longitudinal studies are required in the future to clarify the causal relation between spiritual growth and the entire WM brain structure. Such studies will help extend healthy life expectancy from a new perspective of personal trait.

## Data Availability Statement

The raw data supporting the conclusions of this article will be made available by the authors, without undue reservation.

## Ethics Statement

The studies involving human participants were reviewed and approved by the Ethics Committees of Tokyo Institute of Technology. The patients/participants provided their written informed consent to participate in this study.

## Author Contributions

YY designed and performed the experiment. MF analyzed the data and had primary responsibility for the final content. MF and KW wrote the first draft of the manuscript. All authors approved the final manuscript.

## Conflict of Interest

YY was employed by PwC Consulting LLC. The remaining authors declare that the research was conducted in the absence of any commercial or financial relationships that could be construed as a potential conflict of interest.

## Publisher’s Note

All claims expressed in this article are solely those of the authors and do not necessarily represent those of their affiliated organizations, or those of the publisher, the editors and the reviewers. Any product that may be evaluated in this article, or claim that may be made by its manufacturer, is not guaranteed or endorsed by the publisher.

## Appendix

The nine-item of the spiritual growth subscale.

1. Feel I am growing and changing in positive ways.
2. Believe that my life has purpose.
3. Look forward to the future.
4. Feel content and at peace with myself.
5. Work toward long-term goals in my life.
6. Am aware of what is important to me in life.
7. Feel connected with some force greater than myself.
8. Work toward long-term goals in my life.
9. Expose myself to new experiences and challenges.
